# Recent advances in nanoparticle formulation of oleanolic acid

**DOI:** 10.1186/1749-8546-6-20

**Published:** 2011-05-27

**Authors:** Meiwan Chen, Zhangfeng Zhong, Wen Tan, Shengpeng Wang, Yitao Wang

**Affiliations:** 1 State Key Laboratory of Quality Research in Chinese Medicine, Institute of Chinese Medical Sciences, University of Macau, Macao SAR, China

## Abstract

Oleanolic acid (OA) is a natural triterpenoid possessing anti-inflammatory, antitumor, antiviral, hepatoprotective and antihyperlipidemic effects. Research on the pharmacological activities and clinical applications of OA has made significant progress in the past decade, particularly in the areas such as isolation and purification, chemical modifications, pharmacological research, toxicity studies and clinical use of OA. However, due to its poor aqueous solubility, instability and low bioavailability, OA's clinical applications are still rather limited. Recently, nanoparticulate drug delivery as the biological dimension of nanotechnology has been developed, which may help generate useful formulations of OA for clinical applications. Nanoparticulate drug delivery system enhances the dissolution rate and bioavailability of OA, providing a feasible formulation method for clinical applications.

## Introduction

**Figure 1:**
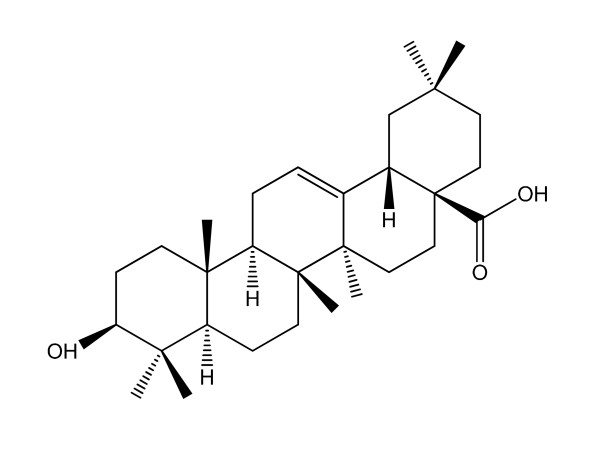
**Chemical structrue of oleanolic acid (OA)**.

Oleanolic acid (OA), a naturally occurring pentacyclic triterpenoid extracted from the leaves and roots of *Olea europaea*, *Viscum album *L., *Aralia chinensis *L. and over 120 other plant species [1], is chemically known as 3β-hydroxy-olea-12-en-28-oic acid [2] (Figure 1). OA exhibits many biological activities such as anti-inflammatory, antitumor, antiviral, hepatoprotective and anti-hyperlipidemic effects. OA has been used in Chinese medicine to treat liver disorders for over 20 years [2]. Conventional formulations of OA are tablets and capsules [3]; however, OA's poor aqueous solubility and low bioavailability *in vivo *make it necessary to develop new formulations for clinical applications.

Derived from nanotechnology, nanoparticulate delivery system provides an innovative approach to drug delivery [4-7]; nanoparticulate technique reduces particles to nanometer ranges, thus reducing the dose and reactive nature of the molecule [8]. Various nanoparticulate drug delivery systems have been explored, such as nanoparticles, nanospheres, nanocapsules, solid lipid nanoparticles (SLN), self-emulsifying drug delivery systems (SEDDS) and submicron/nanoemulsions [9][10]. Compared to conventional dosage forms, nanoparticulate drug delivery system has many advantages, namely enhancement of solubility and stability, protection from toxicity, enrichment of pharmacological activities, improvement of tissue macrophage distribution, bioavailability and sustained delivery, protection from physical and chemical degradation [7,11].

This article reviews recent advances in nanoparticulate formulation of OA.

### Solid lipid nanoparticles

Solid lipid nanoparticles (SLN), which remain solid at room temperature, have emerged as a new pharmaceutical delivery system or formulation to modify the release profile for many drugs [12]. SLN has characteristics of drug carriers such as lipophilicity, hydrophilicity as well as low bio-toxicity. Main advantages of SLN include: controlling drug release, targeting with reduced toxicity, increasing drug stability and high drug payload [13].

High pressure homogenization is an established method for SLN production. Film-ultrasound dispersion technique is another rational and practicable method for developing a new OA injection [9]. A study showed that the OA solid lipid nanoparticles (OA-SLN) by film-ultrasound dispersion technique were with the diameter (62.0 ± 10.3) (mean ± standard deviation) nm, encapsulation efficiency (98.29%), loading rate (8.17%) in OA-SLN [9]. In another study, the researchers prepared OA solid lipid nanoparticles using the optimal preparation conditions (ultrasonic wave time 40 min, OA-phospholipids (1:8), 60 g/L mannitol 15 mL) by film-ultrasonic wave dispersion technique; the appearance of the prepared solid lipid nanoparticles was regular round or ellipse and the diameter distribution was (75 ± 20.3) nm; the envelopment ratio was over 97. 81% [14].

Exploring the protective effect of galactoside - modified OA solid lipid nanoparticles (OA-G10SLN) on CCl_4_-induced acute hepatic injury of rats in an *in vivo *study, Wang *et al. *[15] found that the serum levels of AST and ALT in OA-G10SLN group decreased remarkably compared with a model group, and that the degeneration and necrosis of liver tissues were alleviated significantly, with efficacy better than that in the OA regular solution group.

### Nanosuspension

Nanosuspension technology has been used to increase the solubility, dispersity and homogenization, intravenous injectability, simple production process, universal adaptivity of poorly water soluble drugs [16]. In addition, the formation of suspensions is much more appropriate at low cost and with simple technology to yield a more stable product [16].

There are two major methods for preparing nanosuspension, namely (1) high-pressure homogenization and (2) nanoprecipitation. Homogenization pressure is the major factor determining the average particle size: increased homogenization cycles led to a decreased polydispensible index [17] and surfactants helped keep the system stable [10]. The solubility and dissolution of drug nanoparticles were better than crude drug powder [10]. Researchers obtained OA nanosuspensions with average particle size of 284.9 nm using this method. Drug in the form of spherical or near-spherical nanoparticles in the nanosuspensions showed a faster drug dissolution rate [18]. Pre-treatment of cells with OA nanosuspensions significantly enhanced the hepatoprotective effect against carbon tetrachloride-induced liver injury through lowering serum alanine aminotransferase (ALT) activity and liver malondialdehyde content [18].

In a formulation study [19], several cryoprotectants were employed to study the protective effects of the freeze-dried OA-loaded nanosuspensions. The optimum formulation was selected according to the mean particle sizes of samples before and after the freeze-drying process. The particles of the best sample achieved a mean particle size of 236.3 nm and a much higher polydispersity index of 0.242 [19]. The study showed that the optimum lyophilized powder could be obtained with 10% sucrose as a cryoprotectant.

### Nanocapsules

Loading of drugs into ultrafine host vesicles or colloidal capsules in the nanometer size range was recognized as a technique to optimize controlled drug delivery [20]. Nanocapsules are designed to improve stability, absorption, quantitative tissular transfer and pharmacodynamic activity. Furthermore, they avoided side effects and foreign body irritation with better local and systemic tolerance during and after medication [20].

Dynamic penetration system for sustained OA release from the nanocapsules showed that an HPLC profile curve of the OA loaded nanocapsules fitting the Weibull equation. It was demonstrated that OA loaded nanocapsules sustained the release of OA with a t_1/2 _about 6.7 times of the control [21].

### Liposomes

A liposome is a vesicle consisting of a flexible bilayer and surrounded by an aqueous core domain. Liposomes were used to improve the therapeutic activity and safety of drugs for the past few decades. Advantages of liposomes include high biocompatibility, easy preparation, high chemical versatility and simple modulation of their pharmacokinetic properties by changing the chemical composition of the bilayer components [22].

OA liposomes were prepared with film-ultrasound technique; optimal formulation and preparation techniques were selected through a test of orthogonal design and evaluated according to the entrapment rates and confirmed liposomes [23]. Selected formulation and preparation technique of OA liposomes consistently achieved regular liposomes with an average size of 182 nm and entrapment rate of 92.91%. Chen *et al. *prepared OA liposomes using ethanol injection-sonication and studied the pharmacokinetics of OA liposomes in rats [17]. OA liposomes were almost spherical with a mean diameter of (206.4 ± 4.7) nm. The encapsulation efficiency of OA liposomes was over 90% without hemolyticus. The pharmacokinetic parameters of liposomes were better than those of non-liposomes [17].

### Proliposomes

The concept of proliposome was introduced to improve the stability of liposome. Proliposomes are dry, free-flowing particles that immediately form a liposomal suspension when in contact with water [24]. Proliposome technologies can produce liposome on a large scale and replace the thin film method [25].

A new proliposome preparation method was used to trap OA into the liposomes [26]. Particle size of the liposomes was small and uniformly distributed. The entrapment efficiency was (85.65 ± 7.96) % and increased when pH was increased or the proportion of the the proportion of the drug and the phosphatide (P/D) was increased from 5:1 to 10:1. The liposomes increased the small intestinal absorption of the drug as determined by the isolated small intestinal absorption method, showing a larger area under curve (AUC) in serosal fluid of proliposome than that of the control group [27].

### Self- microemulsifying drug delivery system

Composed of oils and surfactants, self-emulsifying drug delivery systems (SEDDS) was reported to have many advantages, especially in enhancing oral bioavailability of poorly absorbed drugs [28]. Ideal isotropic including co-solvents would disperse in the aqueous environment of the gastrointestinal tract to form a fine oil-in-water emulsion under gentle agitation to improve the oral bioavailability of the drug with poor water-solubility [29]. Compared to conventional emulsions, SNEDDS was reported to be a thermodynamically and physically stable formulation with high solubility and offer an improvement in dissolution rates and extents of absorption, resulting in more reproducible blood-time profiles [30].

Recently, OA SNEDDS was formulated with Sefsol 218, Cremophor EL, Labrasol, and Transcutol P by pseudo-ternary phase diagrams to identify self-emulsification regions for the rational design. A remarkable increase in dissolution was observed for the SNEDDS in comparison with the commercial tablet. Oral absorption of OA from SNEDDS showed a 2.4-fold increase in relative bioavailability. An increased mean retention time of OA in rat plasma was also observed [31]. These results suggest the potentials of SNEDDS in improving dissolution and oral bioavailability for poorly water-soluble triterpenoids. Another study reported the preparation of OA self-microemulsion with ethyl oleate/EL-40/alcohol self-microemulsion system and quality evaluation of OA self-microemulsion with the morphology, particle, diameter distribution, physico-chemical properties and stability [32]. The microemulsion was clear and transparent. The microemulsion vesicles appeared as spherical liquid droplets with a Transmission electron microscopy (TEM) after diluted with average diameter of 49.8 nm. Properties of the microemulsion were stable in the stability test. The authors concluded that the self-microemulsion which improved solubility was easy to prepare. *In vitro *dissolution and absorption kinetics of OA self-microemulsion were studied with paddle method and *in situ *perfusion method respectively. Dissolution of OA was significantly increased by self-microemulsifying drug delivery system compared with commercially available tablets [6]. OA self-microemulsifying system significantly enhanced the absorption of OA in the gastrointestinal tract and improved its bioavailability [33].

### Submicron emulsions

Submicron/nano emulsions are a system of at least two nearly immiscible fluids dispersing one into another in the form of droplets with diameter well below the micron level [34]. Nano/submicron emulsions has drawn much attention from the pharmaceutical, cosmetic and food industries [35]. Submicron/nano emulsions are expected to improve uptake efficiency of lipophilic substances as particle absorption rates in the gastrointestinal tract were correlated to the droplet size [35]. This technology provides colloidal drug carriers for various therapeutic applications such as parenteral, oral, ophthalmic or transdermal delivery systems [36]. Zhao *et al. *developed and validated a simple yet robust HPLC method for the quantitative determination of OA content and partition coefficient of OA in a submicron emulsion-based formulation [37].

## Conclusion

Nanoparticulate drug delivery system enhances the dissolution rate and bioavailability of OA, providing a feasible formulation method for clinical applications.

## Abbreviations

OA: oleanolic acid; SLN: solid lipid nanoparticles; SEDDS: self-emulsifying drug delivery systems; PI: polydispensible index; P/D: the proportion of the drug and the phosphatide; TEM: transmission electron microscopy; OA-SLN: OA solid lipid nanoparticles

## Competing interests

The authors declare that they have no competing interests.

## Authors' contributions

MC and ZZ drafted the manuscript. WT and SW coordinated and revised the study. YW reviewed and confirmed this paper. All authors read and approved the final version of the manuscript.
